# Performance of the three-dimensional laser scanning method to monitor the moisture content of similar material models

**DOI:** 10.1038/s41598-022-18541-w

**Published:** 2022-08-24

**Authors:** Jianfeng Zha, Xicong Yang, Huaizhan Li, Mohan Yang, Chongwu Zhong, Kun Song

**Affiliations:** 1 Engineering Research Center of Mine Ecological Restoration, Ministry of Education, China University of Mining and Technology, No.1 University Road, Xuzhou, 221116 China; 2 Nantun Coal Mine, Yanzhou Coal Industry Co. LTD., Zoucheng, 273515 China; 3 Yunhe Coal Mine, Jining Mining Industry Group Co. LTD., Jining, 272000 China

**Keywords:** Energy science and technology, Engineering

## Abstract

In mining safety and other fields, similar material simulation is the main research method to study the movement and deformation of rock formation and ground surface. However, the inaccurate subsidence laws could be obtained because the strength of the composition materials like gypsum and lime is easily affected by moisture. Therefore, it is crucial to monitor the moisture content when carrying simulation experiments. This paper discussed the feasibility of indirectly measuring the moisture content of similar material models using the three-dimensional (3D) laser scanning reflection intensity through three experiments on similar material specimens. The results showed that the laser reflection intensity was sensitive to the moisture content, incidence angle, and distance with three different relationships and the influence of the two factors could be weakened through the established correction models. However, it was recommended restricting the incidence angle to less than 20° and setting the distance from 4 to 10 m to reduce the complexity of correction. The accuracy of this method reached 1.1% under the monitoring condition of 4 m and the normal incidence, which could meet the requirements for monitoring the moisture content of similar material models. The research results of the paper provide a new method to monitor the moisture content in similar material models.

## Introduction

Underground coal mining could lead to the movement and destruction of overlying rocks, resulting in a large area of surface subsidence and deformation, which would bring a series of environmental and geological issues^1^. Therefore, the subsidence induced by mining and its laws should be predicted accurately and studied carefully for alleviating environmental and geological problems. Generally, physical simulation and numerical simulation are the main research methods in the fields of rock formation movement and surface subsidence. And physical simulation has been widely used in this area based on its direct-viewing observation of the internal movement and deformation of rock mass in various continuous and discontinuous simulations^2,3^. The method of similar material simulation, a common physical simulation method, generally uses sand, gypsum, lime, and some other materials to construct models^4^. During the excavation of a model, the movement and deformation in the rock formation and the ground surface of the model are monitored. Based on the established similarity theory between the model and the prototype, the movement and deformation of the prototype could be estimated from the monitoring results. This simulation research method has been applied in many fields such as coal filling mining, overburden failure evaluation, stope pressure, stability of goaf, etc.^5^. However, it should be pointed out that gypsum and lime, which are usually used as the cement of models, are air-hardening materials whose strength is influenced by the moisture content in the air. Hence, it is inevitable that the strength of the model composed of gypsum, lime, and sand might change due to the variation of moisture content^6,7^. Under natural conditions, the water in the model would gradually evaporate, which could make the strength gradually increase and even largely deviate from the designed strength. At this time, the correlation between the model and the prototype does not accord with the established similarity theory, which would lead to the wrong results if the unsuitable model is used to carry out the simulation study of rock formation.

Data from the study^8^ suggested that the strength of a mixture of sand, lime, and gypsum was directly related to its moisture content. When the moisture content was controlled within a certain range, the strength of the mixture was stable. Therefore, it is necessary to control the moisture content when conducting the model experiments. On the one hand, the moisture content should be confined within the appropriate range; on the other hand, an advanced warning is required to let experimenters know that the strength of the model has deviated from that of the originally designed model when the moisture content of the model exceeds the range.

Methods for monitoring the moisture content of mixtures that consist of sand, gypsum, and lime can be generally divided into contact measurement methods and non-contact measurement methods. The contact measurement methods mainly include drying method^9^, neutron method^10^, time-domain reflectometry (TDR) method^11–13^, frequency-domain reflectometry (FDR) method^14,15^, distributed optical fiber sensing technology method^16,17^, etc.; the non-contact measurement methods mainly include near-infrared method^18^ and microwave measurement method^19^, and others. However, the contact measurement mostly belongs to the point measurement with a small measurement range and could cause certain damage to models, while the non-contact measurement has significant advantages on measuring the entire models with little disturbance to the models themselves.

The three-dimensional (3D) laser scanning method, a non-contact measurement method using near-infrared light, has significant advantages in monitoring the moisture content of a model. For example, a 3D laser scanner can simultaneously measure the displacement field of the model after mining and the moisture content of the model. The basic principle of the method to measure the moisture content is to use the physical mechanism that the laser reflection intensity is significantly lower than the laser emitted intensity since the laser energy is absorbed by the moisture in the model. Specifically, the material with higher moisture content has a much stronger absorption of the laser energy, which could cause the lower reflection intensity of the laser and vice versa. The method of monitoring the moisture content using a 3D laser scanner is currently used in monitoring the moisture content of ancient buildings, vegetation, and energy source^20–22^. In addition, Junttila et al.^23^ and Zhu et al.^24^ have retrieved the moisture content of plants utilizing the laser reflection intensity by the fitting model created by the laser reflection intensity and the moisture content. However, it is still necessary to correct the laser reflection intensity value to weaken the effects of incidence angle and distance before retrieving^23^. Although the accuracy of the intensity value is related to many factors such as material composition, color, surface texture and roughness, incidence angle, distance^25–28^, and some others, the incidence angle and distance are the main influencing factors as far as a specific reflective medium is concerned^29^.

Therefore, there are still many uncertainties about applying this method to the moisture content monitoring of similar material models, which include: (1) whether there exists a significant correlation between the laser reflection intensity and the moisture content of a similar material model; (2) whether incidence angles and distances have disturbance to the sensitivity of the laser reflection intensity to the moisture content; (3) whether the laser reflection intensity can be corrected to the same standard for comparison because the incidence angle and distance of the laser beam corresponding to each scan point are different in actually periodic observation^30^ and (4) whether the accuracy of the method could meet the monitoring requirements on the moisture content of similar material models.

To ascertain the above problems, the similar material specimens are investigated through three experiments in this paper to (1) explore the relationship between the laser intensity and the moisture content for determining whether the laser intensity is sensitive to the moisture content change; (2) study the influence of incidence angle and distance on the laser intensity to find the correction method and give some suggestions about the suitable monitoring position when using 3D laser scanning and (3) discuss the accuracy of this method. Based on the results, we discuss the feasibility of using a 3D laser scanner to monitor the moisture content of similar material models.

## Materials and methods

### Samples and equipment

**Figure 1:**
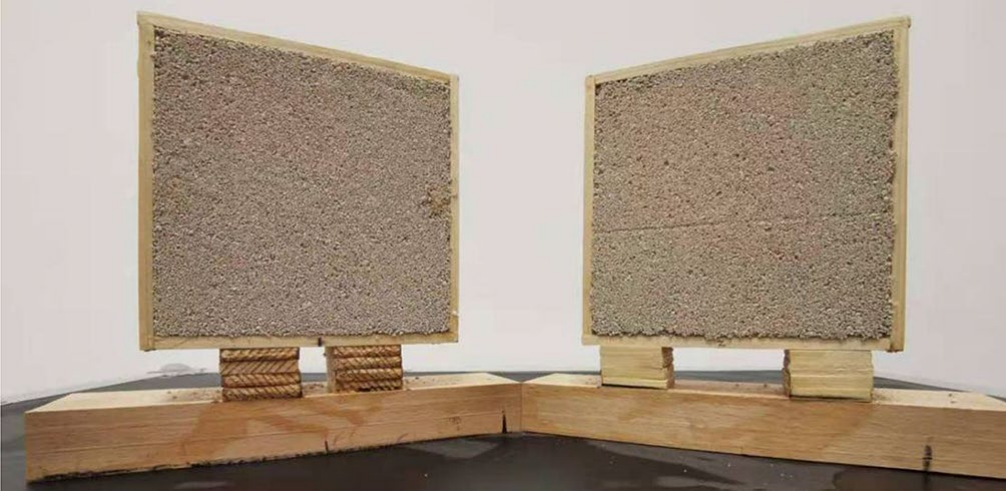
Two displayed specimens.

Gypsum and calcium carbonate were selected as cement and sand was used as aggregate for making the specimens. The proportion of materials (see Table 1) was designed based on He et al.^31^. A total of 10 specimens with a side length of 12 cm and a thickness of 3 cm were made according to the ratio. Some specimens were shown in Fig. 1. During the experiments, the environment ambient temperature and humidity were respectively kept at 4 °C and 70% to prevent the moisture content in specimens from evaporating too quickly^6^. The REIGL VZ1000 3D laser scanner was used to monitor the moisture content in these experiments.

**Table 1:** Proportion of similar material specimens.

Proportion (sand:plaster:calcium carbonate)	Sand (kg)	Plaster (kg)	Calcium carbonate (kg)	Water (ml)	Number of specimens
82:9:9	2.46	0.27	0.27	300	10

### Procedures and data processing

In this paper, the three experiments were designed to (1) evaluate the sensitivity from the relationships between the laser reflection intensity and the moisture content of the specimens; (2) explore the composite correlation of the laser reflection intensity and two factors and how to correct it and (3) study the accuracy of this method by comparing the retrieved moisture content with the results measured by the drying method^9^.

#### Sensitivity experiment

To begin this process, a specimen was scanned at 4 m and the normal incidence with a measurement interval of 24 h and subsequently scanned at incidence angles from 20° to 80° in steps of 20° with an interval of 48 h^6^, while other three specimens were scanned at normal incidence with a measurement interval of 48 h at 4, 6, 8, and 10 m, respectively.

Every specimen was immediately weighed and correspondingly recorded after each scanning. After the whole scanning, all specimens were dried and their moisture contents during each scan were calculated by the drying method.

The original laser reflection intensity of the obtained point cloud belonging to the area of similar material specimens was extracted and output by using the supporting Riscan software.

The original laser reflection intensity is the ratio, which is mostly below zero, of the echo amplitude of the scanned target to that of the white plane Lambertian at the same distance and the normal incidence angle under the extended target conditions^30^. To simplify the calculation, the obtained original laser reflection intensity was normalized to between 0 and 1 according to

1 $$y \, = {1}0^{{x/{1}0}} ,$$

where *x* is the original laser reflection intensity output by Riscan software and *y* is the normalized laser reflection intensity.

After the conversion, the average normalized intensity was regarded as the laser reflection intensity of each scanning and the fitting model between the laser reflection intensity and the moisture content of the specimens was built and analyzed.

#### Incidence angle and distance experiment

**Figure 2:**
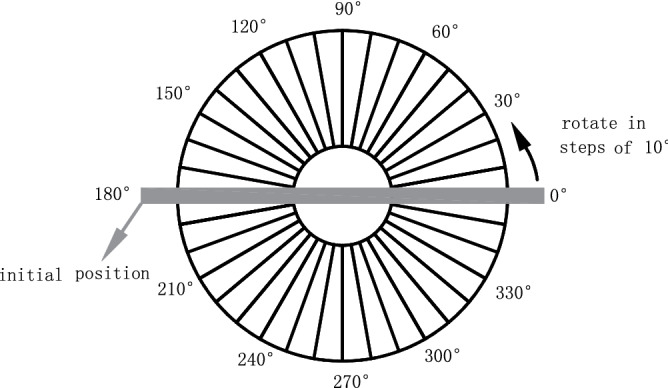
Schematic diagram of rotating (Software: AutoCAD Application 2020; https://www.autodesk.com.cn/).

The second experiment mainly studied the laws of laser reflection intensity responding to the incidence angle and distance change of the specimens at two moisture contents which were calculated as the process in the first experiment. For an obvious comparison, the two moisture contents were less than 2% and higher than 4% respectively. The specimens were placed at measuring distances of 2 m, 4 m, 6 m, 8 m, and 10 m, respectively. At each distance, they were rotated in steps of 10° according to the paper (see Fig. 2) from 0° to 80° during the monitoring.

The process of receiving the returned laser pulse follows the radar range equation, which shows the relationship among the received power, the incidence angle and distance^26^, as follows:

2 $${\text{P}}_{{\text{r}}} = {\text{ C}}\rho {\text{cos}}\theta \, /{\text{ R}}^{{2}} ,$$

3 $${\text{C }} = {\text{ P}}_{{\text{t}}} {\text{D}}_{{\text{r}}}^{{2}} \eta_{{{\text{sys}}}} /{4,}$$

where *P*_*r*_ is the received laser power, *P*_*t*_ is the transmitted laser power, *D*_*r*_ is the receiver aperture diameter, *η*_*sys*_ is the optical system transmission coefficient of radar system, *ρ* is the average reflection coefficient of the extended target, *R* is the distance and *θ* is the incidence angle^26,32^.

Because the receiving method of laser pulse of 3D laser scanner is similar to that of radar system, the equation could be used to describe the receiving process of laser pulse of 3D laser scanner. Inside the receiver, the received laser power is transformed to the laser intensity, so it can be considered that there is a functional relationship between them^32^, as follows

4 $$I \, = \, f \, \left( {P_{r} } \right) \, = \, f \, \left( {\theta , \, R} \right).$$

But this relationship is different in different receiving systems. Assuming that the transformation model is a polynomial model^26,30,32^, the composite relationships were established among the laser reflection intensity and the incidence angle as well as the distance of the specimens at two different moisture contents, they were expressed as

5 $$I \, = \, \lambda_{{1}} + \, \lambda_{{2}} R \, + \, \lambda_{{3}} \theta \, + \, \lambda_{{4}} R^{{2}} + \, \lambda_{{5}} \theta^{{2}} .$$

Then, based on the selected normalization standard, the correction models were also established to correct the laser intensity data^33^. Their basic forms were:

6 $$I \, / \, I_{0} = \, f \, \left( {\theta , \, R} \right),$$

7 $$I_{cor} = \, I \, / \, C,$$

where *θ* is the radians of incidence angles, *R* is the distances, *I* is the laser reflection intensity at different incidence angles and distances before correction, *I*_*0*_ is the selected standard intensity of the standard incidence angle and distance, *I*_*cor*_ is the corrected laser intensity of different incidence angles and distances, *f*(*θ*,* R*) is the established correction function, *C* is the correction coefficient obtained from *f*(*θ*,* R*).

#### Accuracy experiment

To study the accuracy of the method, the moisture contents obtained through laser scanning were compared with the actual moisture contents of each specimen. The incidence angle and distance were set at 0° and 4 m. After each scan, the specimen was also weighed and the subsequent process was the same as that in the first experiment.

**Figure 3:**
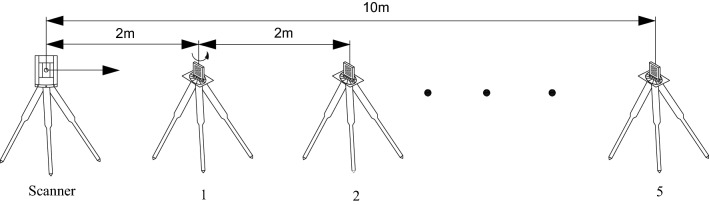
Schematic diagram of monitoring (Software: AutoCAD Application 2020; https://www.autodesk.com.cn/).

The ambient temperature and humidity of the three experiments were the same, while the scanning method (see Fig. 3) was different. The designed scanning incidence angles and distances of the three experiments were shown in Table 2.

**Table 2:** Design of monitoring method of the total three experiments.

Group	Incidence angle (°)	Distance (m)	Moisture content (%)	Number of specimens	Intervals of scanning (h)	Temperature (°C)	Humidity (%)
1	0	4	Change	1	24	4	70
	20,40,60,80	4	Change		48	4	70
	0	6	Change	3	48	4	70
	0	8	Change		48	4	70
	0	10	Change		48	4	70
2	0,10,20,,,80	2	Less than 2%	5	–	4	70
	0,10,20,,,80	4			–	4	70
	0,10,20,,,80	6			–	4	70
	0,10,20,,,80	8			–	4	70
	0,10,20,,,80	10			–	4	70
	0,10,20,,,80	2	Higher than 4%		–	4	70
	0,10,20,,,80	4			–	4	70
	0,10,20,,,80	6			–	4	70
	0,10,20,,,80	8			–	4	70
	0,10,20,,,80	10			–	4	70
3	0	4	Change	1	24	4	70

According to the fitting model built in the first experiment, the moisture contents of the specimen were retrieved from its laser reflection intensity and compared with its actual moisture contents calculated by the drying method. The difference of the data obtained through two different measurement methods was used to calculate the RMSE based on Bessel’s formula and the RMSE was utilized to describe and evaluate the accuracy of the 3D laser scanning method under the monitoring condition of 4 m and the normal incidence.

## Results

### The relationships between laser reflection intensity and moisture content

**Figure 4:**
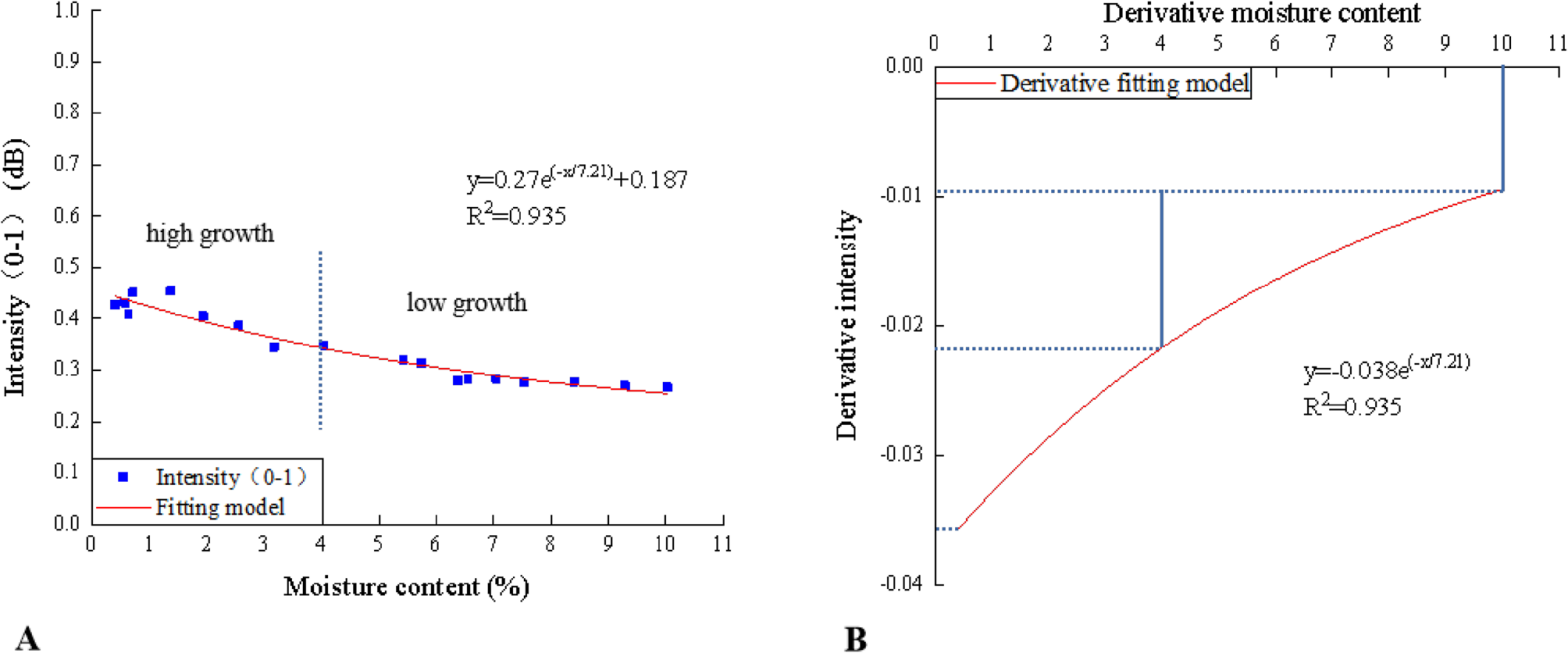
Fitting (**A**) and derivative (**B**) relationships between the laser reflection intensity (*y*) and the moisture content (*x*) of the specimen at 4 m with the normal incidence angle (Software: Riscan pro 2.1; riscan-pro.software.informer.com; Origin 2021b; https://www.originlab.com/index.aspx?go=PRODUCTS/Origin).

The correlation between the laser reflection intensity and moisture content was studied based on the specimen monitored at 4 m with the normal incidence angle using the 3D laser scanner. In Fig. 4 there was a clear trend of decreasing the moisture content, the value of the laser reflection intensity gradually increased exponentially. Further analysis showed that the intensity reached the maximum when the moisture content was close to 0%. Besides, there was a significantly different changing trend of the intensity. The laser reflection intensity value had a higher rate of growth when the moisture content was lower than 4%.

The derivative relationship showed this difference more clearly. From the chart, it could be seen that the absolute intensity change rate was from 0.02 to around 0.04 when the moisture content was less than 4%, while it dropped from 0.02 to 0.01 when the moisture content exceeded 4%. The results, as shown in the derivative fitting model, also indicated that the absolute value of the intensity change rate increased nonlinearly with the moisture content decreasing and still existed even the moisture content has been to 10%.

**Figure 5:**
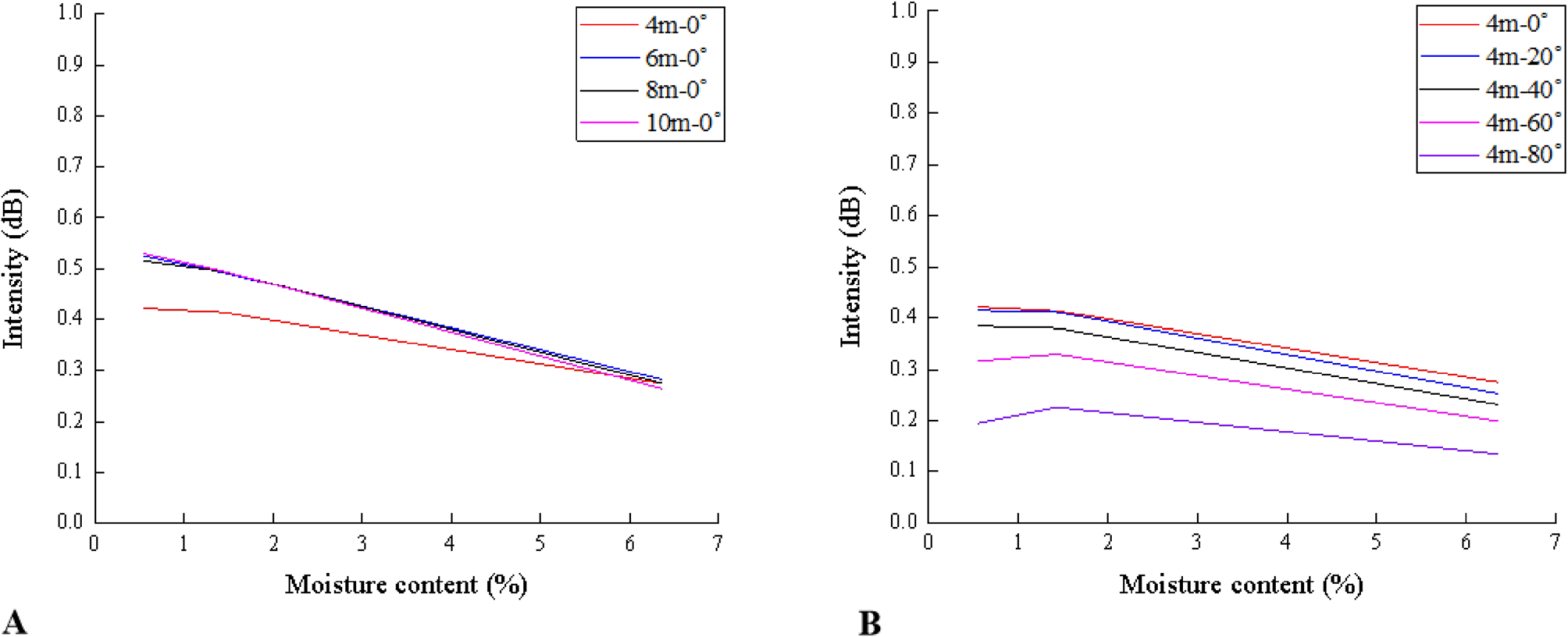
Relationships between the laser reflection intensity and the moisture content of the specimens with the normal incidence angle (**A**) and with the distance of 4 m (**B**) (Software: Riscan pro 2.1; riscan-pro.software.informer.com; Origin 2021b; https://www.originlab.com/index.aspx?go=PRODUCTS/Origin).

The results of the correlational analysis among the intensity and moisture contents, including 0.55%, 1.43%, and 6.35%, were shown in Fig. 5. It was apparent from the first chart in Fig. 5 that the relationships under the same angle at different distances were similar, especially 6 m, 8 m, and 10 m. And another illustration also showed the similarity of intensity change rate under the same distance at different angles.

### The relationships among laser reflection intensity and incidence angle and distance

**Figure 6:**
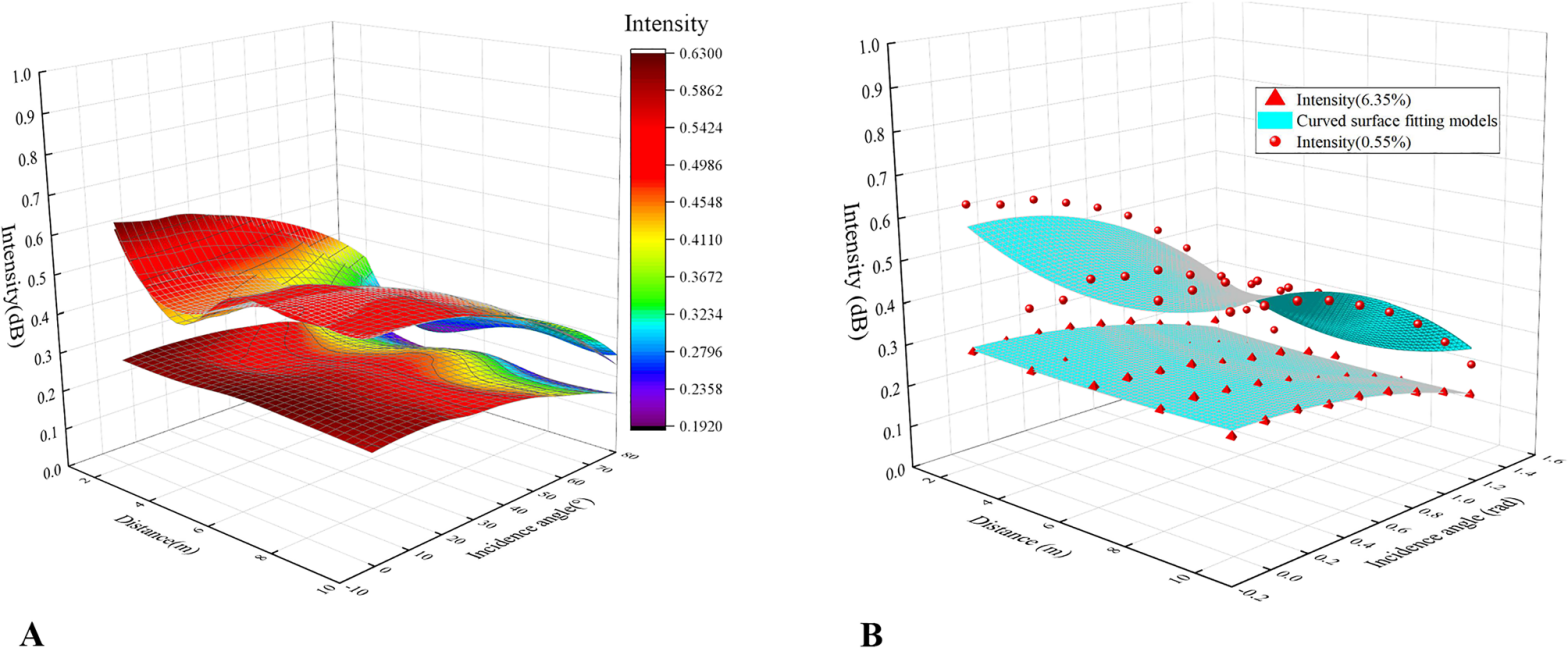
Composite relationships (**A**) and fitting functions (**B**) among the laser reflection intensity and incidence angle as well as distance at the moisture contents of 0.55% and 6.35% (Software: Riscan pro 2.1; riscan-pro.software.informer.com; Origin 2021b; https://www.originlab.com/index.aspx?go=PRODUCTS/Origin).

Figure 6 showed the composite relationships and the curved surface fitting models among the laser intensity and the incidence angle and distance of the specimens under 0.55% and 6.35% moisture contents and the parameters were shown in Table 3. It was obvious that the composite relationships at the two moisture contents were different. The laser reflection intensity value of 0.55% moisture content was higher than that of 6.35% moisture content at the same angle and distance.

**Table 3:** Parameters and their evaluation of the curved surface fitting models of the composite relationships among the laser reflection intensity and incidence angle as well as distance at the two moisture contents.

Moisture content (%)	Parameters					
	λ_1_ (dB)	λ_2_ (dB/m)	λ_3_ (dB/rad)	λ_4_ (dB/m^2^)	λ_5_ (dB/rad^2^)	R^2^
0.55	0.676	− 0.056	0.028	0.005	− 0.159	0.845
6.35	0.291	− 0.006	0	0	− 0.061	0.955

**Figure 7:**
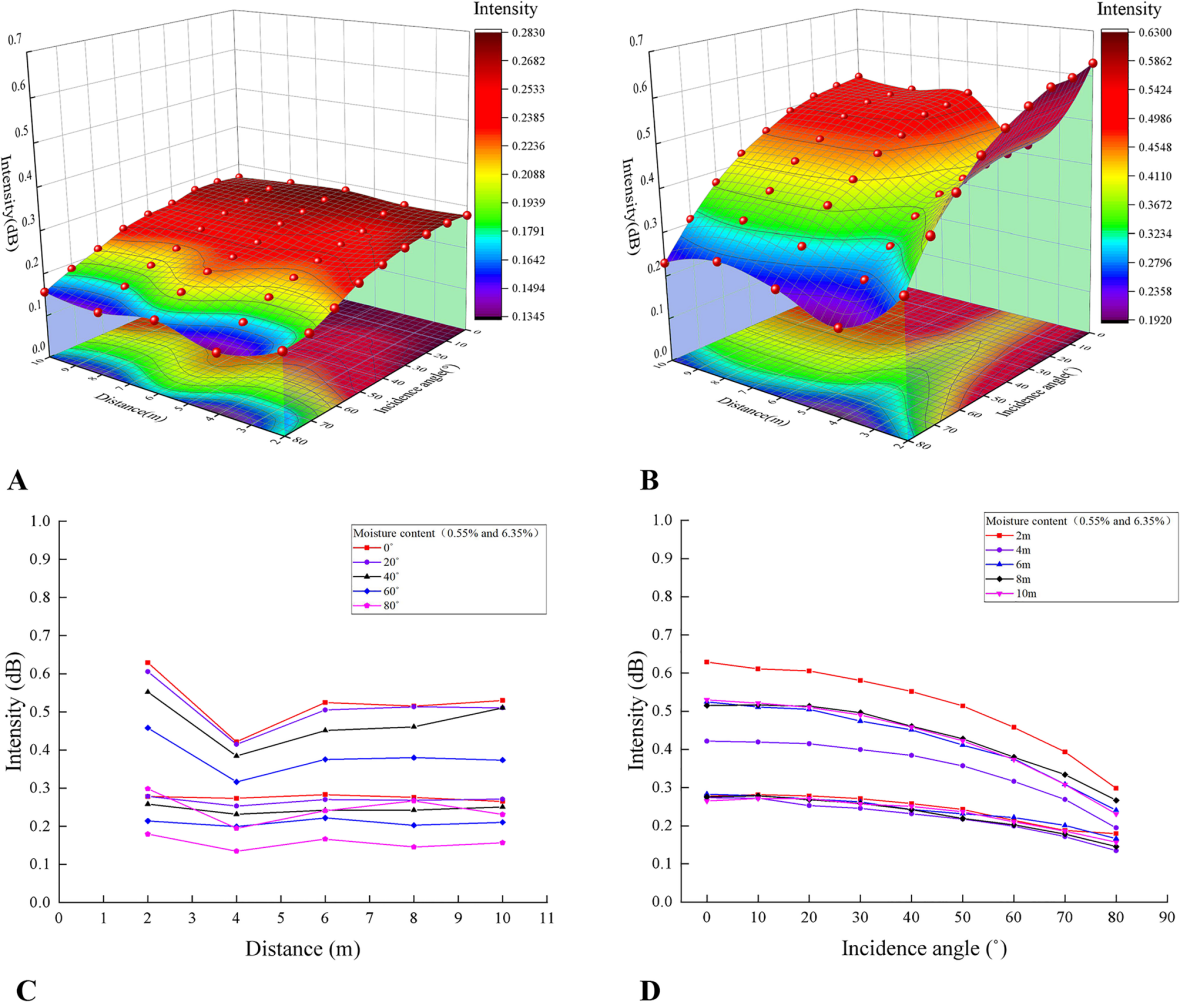
Composite relationships among the laser reflection intensity and incidence angle as well as distance at the moisture contents of 0.55% and 6.35% (**A**, **B**) and their projection relationships at the same incidence angle and distance (**C**, **D**) (Software: Riscan pro 2.1; riscan-pro.software.informer.com; Origin 2021b; https://www.originlab.com/index.aspx?go=PRODUCTS/Origin).

Figure 7 provided more details about the composite relationships. The most interesting aspect of the graphs was that the laser intensity value mostly had a reduction at the distances of 4 m and 6 m to some extent, as shown in the two surface diagrams in Fig. 7.

The above could be seen more intuitively from the first line chart. From this chart, the change in laser reflection intensity between 2 and 4 m was greater than that from 4 to 10 m, showing a significant characteristic of a piecewise function. In addition, with the moisture content falling, the distance had a significantly stronger effect on the intensity of the same incidence angle.

The last illustration in Fig. 7 showed that (1) the relationship was that the reflection intensity value decreased nonlinearly with the increasement of the incidence angle but this change was less obvious in the range of 0° to 20°; (2) when the moisture content was 0.55%, the intensity change rate was relatively larger than that of the 6.35% moisture content.

The above data obtained under the two moisture contents were corrected to different standards, including 4 m, 6 m, and 8 m under the normal angle, through the established correction models. The parameters of the correction formulae with different normalization standards were shown in Table 4.

**Table 4:** Fitting parameters and their evaluation of the established correction models based on different standards.

Standard	Moisture content (%)	Parameters					
		λ_1_	λ_2_ (m^−1^)	λ_3_ (rad^−1^)	λ_4_ (m^−2^)	λ_5_ (rad^−2^)	R^2^
4 m–0°	0.55	1.612	− 0.144	0.056	0.011	− 0.367	0.820
	6.35	1.115	− 0.031	− 0.042	0.002	− 0.232	0.960
6 m–0°	0.55	1.295	− 0.116	0.045	0.009	− 0.295	0.820
	6.35	1.077	− 0.030	− 0.040	0.002	− 0.225	0.960
8 m–0°	0.55	1.320	− 0.118	0.046	0.009	− 0.301	0.820
	6.35	1.106	− 0.031	− 0.041	0.002	− 0.231	0.960

**Figure 8:**
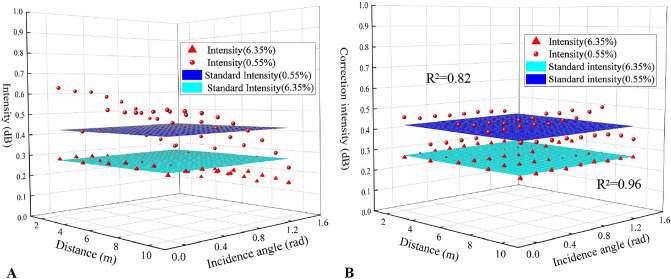
Intensity value before correction (**A**) and after correction (**B**) with the standard intensity of the normal incidence angle and 4 m (Software: Riscan pro 2.1; riscan-pro.software.informer.com; Origin 2021b; https://www.originlab.com/index.aspx?go=PRODUCTS/Origin).

**Figure 9:**
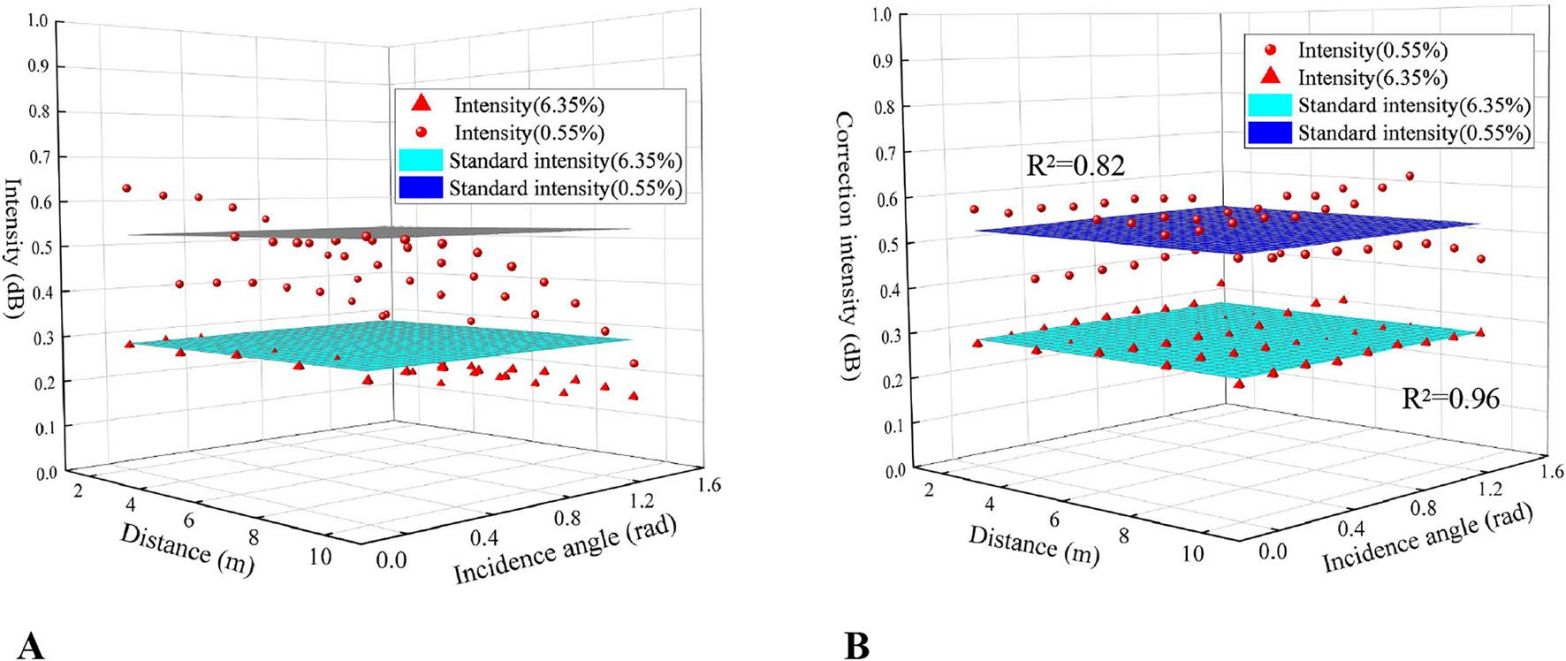
Intensity value before correction (**A**) and after correction (**B**) with the standard intensity of the normal incidence angle and 6 m (Software: Riscan pro 2.1; riscan-pro.software.informer.com; Origin 2021b; https://www.originlab.com/index.aspx?go=PRODUCTS/Origin).

**Figure 10:**
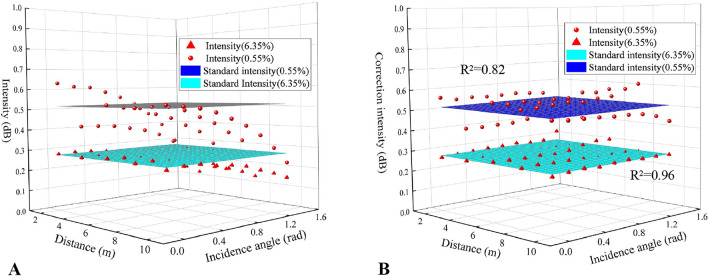
Intensity value before correction (**A**) and after correction (**B**) with the standard intensity of the normal incidence angle and 8 m (Software: Riscan pro 2.1; riscan-pro.software.informer.com; Origin 2021b; https://www.originlab.com/index.aspx?go=PRODUCTS/Origin).

Figures 8, 9, and 10 respectively showed the corrected results based on different standard intensity values. It could be seen from the graphs that the corrected intensity value was closer to the selected standard surface to a certain extent and also more concentrated compared with the previous distribution.

Taking the standard intensity values as the true values, the root-mean-square errors (RMSEs) of the corrected laser reflection intensity were shown in Table 5. As it showed, though the RMSEs were all less than 0.5 dB, the RMSEs of the 0.55% moisture content were significantly greater than that of 6.35%.

**Table 5:** Performance of the correction models as evaluated by the RMSEs.

Standard				RMSE (dB)
Distance (m)	Incidence angle (°)	Moisture content (%)	Intensity (dB)	
4	0	0.55	0.42	0.043
		6.35	0.27	0.014
6	0	0.55	0.52	0.054
		6.35	0.28	0.014
8	0	0.55	0.52	0.372
		6.35	0.28	0.014

### Accuracy of 3D laser scanning method

It could be seen from the data in Table 6 showed the difference between the moisture content retrieved from the intensity and the moisture content measured by the drying method, whose minimum and maximum absolute values were 0.10% and 1.84%, respectively. The RMSE of the 3D laser scanning method to monitor the moisture content of a similar material specimen was 1.1%.

**Table 6:** The differences between the retrieved moisture content and the actual moisture content of the specimen.

Number of scanning	Retrieved moisture contents (%)	Actual moisture contents (%)	Differences
1	1.40	0.30	− 1.10
2	0.91	0.42	− 0.49
3	1.51	0.48	− 1.03
4	1.09	0.55	− 0.54
5	1.33	1.43	0.10
6	4.50	3.39	− 1.11
7	7.89	6.16	− 1.73
8	8.19	6.35	− 1.84

## Discussion

To evaluate the performance of using 3D laser scanning to monitor similar material specimens, we analyzed the feasibility of the monitoring method based on three experiments from the three aspects of sensitivity, influencing factors, and accuracy, respectively.

In the changing process of the specimen from the highest moisture content to the lowest moisture content, the laser intensity increased exponentially in Fig. 4, which accords with the findings of Zhu et al.^24^, indicating that the laser intensity is sensitive to the change of moisture content during the monitoring process. Therefore, for some similar material models designed under about 10% moisture content^5,6,34,35^, 3D laser monitoring could maintain sensitivity during the process of moisture content′s gradual loss.

In addition, the relationship between the laser intensity and the moisture content demonstrated that when the moisture content was less than 4%, the laser intensity had a significantly greater response to the moisture content in Fig. 4. It is noticeable that most simulation experiments are carried out when the moisture content of a similar material model is between 2 and 4% because its mechanical properties are relatively stable in this section^36^. Therefore, in the optimum observation interval, this high sensitivity is relatively beneficial for monitoring the moisture content change. Moreover, this sensitivity would not be significantly affected by the incidence angle and distance, as proved by the fact that the similar relationships between the moisture content and the laser intensity at different positions (see Fig. 5).

From the above, for most of the similar material models, the change of moisture content could be detected using 3D laser scanning.

The laser reflection intensity at the same angle and distance of 0.55% moisture content was higher than the intensity of 6.35% moisture content in Fig. 6. A possible explanation for this might be that the laser energy is absorbed by excessive moisture. As the moisture decreases, the reflectivity of the specimen surface increases due to the growing laser energy^24^. Correspondingly, the relationships among laser reflection intensity, incidence angle, and distance would be diverse under different moisture contents (see Fig. 6).

The relationships of the two influencing factors and the laser reflection intensity under the two moisture contents in Fig. 7 were also reported by Tan et al.^30^. However, there existed an obvious feature of the relationships between the intensity and distance, a large attenuation at 4 m in the two surface diagrams of Fig. 7, which may mainly be caused by the optical defocusing effect and the non-overlapping field of view of the laser receiver at a proximity distance^29^, while the small fluctuations at 6 m might be due to the errors. And the close-range effect turned to be greater as the moisture content dropped (see Fig. 7). Zhu et al.^24^ reported that the laser intensity of the material with higher reflectivity had a greater attenuation as the incidence angle increased comparing with the material with lower reflectivity. Similarly, we also found that the effect of incidence angle and distance on the laser intensity increased as the moisture content decreased (see Fig. 7).

Therefore, the influence of the incidence angle and distance would be diverse due to the different moisture contents, which could lead to the different composite relationships among the incidence angle, distance, and intensity under different moisture contents, and then the laser intensity correction models established from the composite relationships are also different (see Table 4)^30^. It would be more complicated for the correction to the intensity of the point clouds in different positions of similar material models with different moisture contents, which can also be inferred from the calibration process. Although the corrected intensity value is closer to the standard value, the workload of correcting is too complex after each real-time monitoring.

For reducing the complexity of correcting, we recommend that the incidence angle should be within 20˚ and the distance should be controlled from 4 to 10 m when using a scanner to monitor similar material models. This is because Fig. 7 showed that the laser intensity changes in this range were not significant. Besides, the reason why the relative distance is preferably greater than 4 m is due to the proximity effect and it could be set greater than 6 m for better results. The upper limit of distance can be set at about 8 m if the space is not large enough when similar material models are placed indoors. The model could be measured by partition to ensure the incidence angle and distance within the suggested range if the model is too wide. It is considered that the intensity value of the point clouds in this range is approximately unaffected by the incidence angle and distance, which is equal to having been under a similar normalization standard. The correction would not be required, which reduces the workload of correction.

The accuracy of the method was just 1.1% under the monitoring condition of 4 m and the normal incidence. The optimum interval to conduct the simulation experiments was the moisture content of about 2–4%^34^. When the model’s moisture content was lower than 2% or higher than 4%, the difference between the retrieved moisture content and the actual moisture content would be larger (see Table 6). Fortunately, we just needed to monitor the model when its moisture content was in the range of about 2–4% in the actual experiment. When the moisture content was lower than 2% or higher than 4%, the general model was no longer applicable and we did not need to monitor it accordingly. From this point of view, this method was suitable for model’s moisture content monitoring, especially in the optimum interval and the measurement accuracy could be further improved by taking the numerical average of multiple measurements. It should be pointed out that from Table 6, it seemed to exist the systematic error, which was actually caused by the fitting error. For example, some points in Fig. 4a were under the curve. Their true intensity was lower than their fitting intensity of the same actual moisture content (see Fig. 4a). Then when using the fitting formula and the true intensity to retrieve, the retrieved moisture content would naturally be higher than the actual one. In Table 6, most fitted intensity of the measurement points was higher than the measured intensity, which led to the overestimation of the results. The phenomenon was actually caused by the error of this measurement method.

Therefore, the moisture content of the similar material models within about 10% moisture could be determined by the established fitting model between it and intensity under the premise that the incidence angle and distance are within the suggested range. Specifically, it is first necessary to limit the relative position of the 3D laser scanner and the similar material model in the practice. For example, the scanner could be placed in the middle and at a distance of 4 m from the model and the height of the scanning center should be the same as the model center. Then the overall point cloud data of the similar material model could be retrieved to obtain the overall moisture content of the model based on the established relationship between the moisture content and the laser intensity at 4 m and the normal incidence angle. And then based on the relationship between the strength and the moisture content of the model^34^, the strength of the model could be clarified at any time to determine whether it meets the similarity theory and is suitable to carry out the simulation experiments.

## Conclusions

In this paper, 3D laser scanning technology is used as a general data acquisition method to monitor the moisture content of similar material models. We prove the feasibility of this method from three aspects by the experiments of similar material specimens. We have discussed the sensitivity and relationships among the laser reflection intensity, moisture content, incidence angle, and distance. Then, the accuracy of the method has been also calculated. Through a series of experiments, we find that.

1. Though the composite relationships among the reflection intensity and incidence angle and distance of different moisture contents are different, the reflection intensity all decreases nonlinearly with the incidence angle increasing and has obvious piecewise characteristics from 2 to 10 m.
2. For different moisture contents and naturalization standards, different correction models need to be established to weaken the influence of incidence angle and distance. It is recommended that the incidence angle should be within 20° and the distance should be controlled in the range of 4–10 m to reduce the complexity of correction during actually monitoring.
3. The laser reflection intensity gradually increases exponentially with the decrease of moisture content. The laser intensity is sensitive to the moisture content in the process of its evaporation and could even be more sensitive in the optimal monitoring interval for the similar material models of about 10% moisture content, which is hardly affected by the incidence angle and distance. The accuracy of 3D laser scanning is 1.1% under the monitoring condition of 4 m and the normal incidence, which could satisfy the monitoring requirement of the moisture content.

To our knowledge, the above provides new ideas for the laser scanning technology to monitor the moisture content, our findings could be considered to provide a new way to monitor the moisture content of similar material models.

## Supplementary Information

Supplementary Tables.

## Data Availability

The datasets generated during and analysed during the current study are available from the corresponding author on reasonable request.
