# Supplementary material for: Performance of the three-dimensional laser scanning method to monitor the moisture content of similar material models
Source: Sci Rep. 2022 Aug 24;12:14433. doi: 10.1038/s41598-022-18541-w (PMC9402562; doi:10.1038/s41598-022-18541-w)
Supplement: Supplementary file 1 — Supplementary Tables. [file 41598_2022_18541_MOESM1_ESM.pdf]

Performance of the three-dimensional laser scanning method to monitor the moisture content of similar material models

Jianfeng Zha [a, *], Xicong Yang [a], Huaizhan Li [a], Mohan Yang [a], Chongwu Zhong [b], Kun Song [c]

[a] Engineering Research Center of Mine Ecological Restoration, Ministry of Education, China University of Mining and Technology, No.1 University Road, Xuzhou, 221116, China

[b] Nantun Coal Mine, Yanzhou Coal Industry Co. LTD., Zoucheng, 273515, China

[c] Yunhe coal mine, Jining Mining Industry Group Co. LTD., Jining, 272000, China

Table 1 The results of statistical significance of the model coefficients in Fig.4

		Value	Standard error	t value	Probability > t	Correlation
Intensity (0-1)	y0	0.18693	0.06259	2.98666	0.0922	0.99558
	A1	0.27231	0.0562	4.84572	2.13852E-4	0.98622
	t1	7.2136	3.05	2.36511	0.03193	0.98439

Fitting model: y = A1*exp(-x/t1) + y0

Table 2 The results of variance of the fitting model in Fig.4

	Intensity (0-1)
Points	18
Degrees of freedom	15
Reduced Chi-Sqr	3.11565E-4
Residual sum of squares	0.00467
R^2(COD)	0.94267
R^2 (after correction)	0.93503
Fitting results	Successful

Table 3 The results of variance analysis in Fig.4

		Sum of squares	Mean square	F	Probability > F
Intensity (0-1)	Regression	0.07685	0.03842	123.3279	4.87199E-10
	Residual	0.00467	3.11565E-4		

Table 4 The results of statistical significance of the model coefficients in Fig.6 under 0.55 moisture content

		Value	Standard error	t value	Probability > t	Correlation
Intensity (0-1)	z0	0.6759	0.04136	16.34305	4.82215E-19	0.97419
	a	-0.05927	0.01267	-4.67611	3.46039E-5	0.99376
	b	0.02818	0.0625	0.45088	4.94782E-5	0.98363
	c	0.00453	9.92657E-4	4.56111	4.94782E-5	0.9857
	d	-0.1586	0.03728	-4.25467	1.26945E-4	0.96489

Fitting model: $z = z0+ax+by+cx^2+dy^2$

Table 5 The results of variance of the fitting model in Fig.6 under 0.55 moisture content

	Intensity (0-1)
Points	45
Degrees of freedom	39
Reduced Chi-Sqr	0.00199
Residual sum of squares	0.07747
R^2(COD)	0.84511
R^2 (after correction)	0.82525
Fitting results	Successful

Table 6 The results of variance analysis in Fig.6 under 0.55 moisture content

		Sum of squares	Mean square	F	Probability > F
Intensity (0-1)	Regression	0.42272	0.08454	42.55901	8.98117E-15
	Residual	0.07747	0.00199		

Table 7 The results of statistical significance of the model coefficients in Fig.6 under 6.35 moisture content

		Value	Standard error	t value	Probability > t	Correlation
Intensity (0-1)	z0	0.29121	0.00872	33.3911	2.65249E-30	0.97419
	a	-0.0056	0.00267	-2.10519	0.04177	0.99376
	b	-3.0E-4	0.01318	-0.02297	0.98179	0.98363
	c	4.22E-4	2.09329E-4	2.01836	0.05047	0.9857
	d	-0.061	0.00786	-7.7542	2.02079E-9	0.96489

Fitting model: $z = z0+ax+by+cx^2+dy^2$

Table 8 The results of variance of the fitting model in Fig.6 under 6.35 moisture content

	Intensity (0-1)
Points	45
Degrees of freedom	39
Reduced Chi-Sqr	8.83381E-5
Residual sum of squares	0.00345
R^2(COD)	0.95458
R^2 (after correction)	0.94875
Fitting results	Successful

Table 9 The results of variance analysis in Fig.6 under 6.35 moisture content

		Sum of squares	Mean square	F	Probability > F
Intensity (0-1)	Regression	0.0724	0.01448	163.9198	4.36403E-25
	Residual	0.00345	8.83381E-5		

Table 10 The results of statistical significance of the model coefficients in Table 4 under 0.55 moisture content of 4 m, 0 angle

		Value	Standard error	t value	Probability > t	Correlation
I/I0	z0	1.61162	0.07102	22.6913	2.3807E-36	0.97571
	a	-0.1438	0.02121	-6.77861	1.97447E-9	0.99381
	b	0.05612	0.11277	0.49767	0.6201	0.98563
	c	0.01099	0.00165	6.64632	3.51678E-9	0.9857
	d	-0.3673	0.06806	-5.39661	6.91069E-7	0.96821

Fitting model: $z = z0+ax+by+cx^2+dy^2$

Table 11 The results of variance of the fitting model in Table 4 under 0.55 moisture content of 4 m, 0 angle

	I/I0
Points	85
Degrees of freedom	79
Reduced Chi-Sqr	0.01041
Residual sum of squares	0.82265
R^2(COD)	0.83081
R^2 (after correction)	0.8201
Fitting results	Successful

Table 12 The results of variance analysis in Table 4 under 0.55 moisture content of 4 m, 0 angle

		Sum of squares	Mean square	F	Probability > F
I/I0	Regression	4.03956	0.80791	77.58446	4.95637E-29
	Residual	0.82265	0.01041		

Table 13 The results of statistical significance of the model coefficients in Table 4 under 6.35 moisture content of 4 m, 0 angle

		Value	Standard error	t value	Probability > t	Correlation
I/I0	z0	1.11474	0.02038	54.7080	8.45586E-76	0.97721
	a	-0.0314	0.00664	-4.72453	7.62715E-6	0.9939
	b	-0.0416	0.02983	-1.39452	0.16628	0.98438
	c	0.00214	5.38744E-4	3.97148	1.35574E-4	0.98517
	d	-0.2324	0.018	-12.91	6.03915E-23	0.96641

Fitting model: $z = z0+ax+by+cx^2+dy^2$

Table 14 The results of variance of the fitting model in Table 4 under 6.35 moisture content of 4 m, 0 angle

	I/I0
Points	105
Degrees of freedom	99
Reduced Chi-Sqr	9.93729E-4
Residual sum of squares	0.09838
R^2(COD)	0.96202
R^2 (after correction)	0.9601
Fitting results	Successful

Table 15 The results of variance analysis in Table 4 under 6.35 moisture content of 4 m, 0 angle

		Sum of squares	Mean square	F	Probability > F
I/I0	Regression	2.49193	0.49839	501.53	1.2521E-68
	Residual	0.09838	9.93729E-4		

Table 16 The results of statistical significance of the model coefficients in Table 4 under 0.55 moisture content of 6 m, 0 angle

		Value	Standard error	t value	Probability > t	Correlation
I/I0	z0	1.29495	0.05707	22.6913	2.3807E-36	0.97571
	a	-0.1155	0.01704	-6.77861	1.97447E-9	0.99381
	b	0.0451	0.09061	0.49767	0.6201	0.98563
	c	0.00883	0.00133	6.64632	3.51678E-9	0.9857
	d	-0.2951	0.05469	-5.39661	6.91069E-7	0.96821

Fitting model: $z = z0+ax+by+cx^2+dy^2$

Table 17 The results of variance of the fitting model in Table 4 under 0.55 moisture content of 6 m, 0 angle

	I/I0
Points	85
Degrees of freedom	79
Reduced Chi-Sqr	0.00672
Residual sum of squares	0.53112
R^2(COD)	0.83081
R^2 (after correction)	0.8201
Fitting results	Successful

Table 18 The results of variance analysis in Table 4 under 0.55 moisture content of 6 m, 0 angle

		Sum of squares	Mean square	F	Probability > F
I/I0	Regression	2.60803	0.52161	77.58446	4.95637E-29
	Residual	0.53112	0.00672		

Table 19 The results of statistical significance of the model coefficients in Table 4 under 6.35 moisture content of 6 m, 0 angle

		Value	Standard error	t value	Probability > t	Correlation
I/I0	z0	1.07707	0.01969	54.7080	8.45586E-76	0.97721
	a	-0.0303	0.00642	-4.72453	7.62715E-6	0.9939
	b	-0.0402	0.02883	-1.39452	0.16628	0.98438
	c	0.00207	5.20539E-4	3.97148	1.35574E-4	0.98517
	d	-0.2246	0.0174	-12.91	6.03915E-23	0.96641

Fitting model: $z = z0+ax+by+cx^2+dy^2$

Table 20 The results of variance of the fitting model in Table 4 under 6.35 moisture content of 6 m, 0 angle

	I/I0
Points	105
Degrees of freedom	99
Reduced Chi-Sqr	9.27704E-4
Residual sum of squares	0.09184
R^2(COD)	0.96202
R^2 (after correction)	0.9601
Fitting results	Successful

Table 21 The results of variance analysis in Table 4 under 6.35 moisture content of 6 m, 0 angle

		Sum of squares	Mean square	F	Probability > F
I/I0	Regression	2.32636	0.46527	501.53	1.2521E-68
	Residual	0.09184	9.27704E-4		

Table 22 The results of statistical significance of the model coefficients in Table 4 under 0.55 moisture content of 8 m, 0 angle

		Value	Standard error	t value	Probability > t	Correlation
I/I0	z0	1.31934	0.05814	22.6913	2.3807E-36	0.97571
	a	-0.1177	0.01736	-6.77861	1.97447E-9	0.99381
	b	0.04595	0.09232	0.49767	0.6201	0.98563
	c	0.009	0.00135	6.64632	3.51678E-9	0.9857
	d	-0.3007	0.05572	-5.39661	6.91069E-7	0.96821

Fitting model: $z = z0+ax+by+cx^2+dy^2$

Table 23 The results of variance of the fitting model in Table 4 under 0.55 moisture content of 8 m, 0 angle

	I/I0
Points	85
Degrees of freedom	79
Reduced Chi-Sqr	0.00698
Residual sum of squares	0.55132
R^2(COD)	0.83081
R^2 (after correction)	0.8201
Fitting results	Successful

Table 24 The results of variance analysis in Table 4 under 0.55 moisture content of 8 m, 0 angle

		Sum of squares	Mean square	F	Probability > F
I/I0	Regression	2.7072	0.54144	77.58446	4.95637E-29
	Residual	0.55132	0.00698		

Table 25 The results of statistical significance of the model coefficients in Table 4 under 6.35 moisture content of 8 m, 0 angle

		Value	Standard error	t value	Probability > t	Correlation
I/I0	z0	1.10624	0.02022	54.7080	8.45586E-76	0.97721
	a	-0.0311	0.00659	-4.72453	7.62715E-6	0.9939
	b	-0.0413	0.02961	-1.39452	0.16628	0.98438
	c	0.00212	5.34637E-4	3.97148	1.35574E-4	0.98517
	d	-0.2306	0.01787	-12.91	6.03915E-23	0.96641

Fitting model: $z = z0+ax+by+cx^2+dy^2$

Table 26 The results of variance of the fitting model in Table 4 under 6.35 moisture content of 8 m, 0 angle

	I/I0
Points	105
Degrees of freedom	99
Reduced Chi-Sqr	9.78635E-4
Residual sum of squares	0.09688
R^2(COD)	0.96202
R^2 (after correction)	0.9601
Fitting results	Successful

Table 27 The results of variance analysis in Table 4 under 6.35 moisture content of 8 m, 0 angle

		Sum of squares	Mean square	F	Probability > F
I/I0	Regression	2.45407	0.49081	501.53	1.2521E-68
	Residual	0.09688	9.78635E-4		
